# Transforming research recruitment: Leveraging EHR systems and patient portals

**DOI:** 10.1017/cts.2024.692

**Published:** 2024-12-26

**Authors:** Megan Schwinne, Edward Woods, Barney Chan, Candace D. Speight, Vivian Corry, Neal W. Dickert, Gabriel R. Najarro

**Affiliations:** 1 Department of Biomedical Informatics, Emory University School of Medicine, Atlanta, GA, USA; 2 Georgia Clinical and Translational Science Alliance, Emory University School of Medicine, Atlanta, GA, USA; 3 Department of Internal Medicine, Emory University School of Medicine, Atlanta, GA, USA; 4 Division of Cardiology, Department of Medicine, Emory University School of Medicine, Atlanta, GA, USA

**Keywords:** Electronic health records, patient portals, research recruitment, patient participation, health communication

## Abstract

Electronic health records and patient portals are increasingly utilized to enhance research recruitment efficiency, yet response patterns across patient groups remain unclear. We examined 10 studies at Emory Healthcare that used these tools to identify and recruit 24,000 patients over 1 year. Response rates were lower among males and Black individuals, though study interest was higher among respondents. Interest was also greater among those with frequent healthcare interactions and lower comorbidity. In a large academic health system, portal-based recruitment offered a streamlined approach to research recruitment and patient engagement, with minor variations across patient characteristics warranting continued study.

## Introduction

Electronic health records (EHR) have revolutionized medical information storage, offering clinicians a comprehensive and organized view of patient data and increased data accessibility [1]. Electronic patient portals, available in over 90% of healthcare systems [2,3], foster direct engagement between healthcare practitioners and patients [4], in addition to providing patients with greater access to their own data [5,6,7]. Combining EHR data and patient portal systems has emerged as a promising avenue to enhance recruitment for clinical research.

Participant recruitment is a major bottleneck in research, often monopolizing up to 30% of the research timeline and consuming significant resources [8]. Traditional recruitment strategies – such as online and in-person advertising, direct physician–patient interactions, and manual chart reviews – are often inefficient, labor-intensive, and limited in outreach [9]. These limitations have consequences; it has been estimated that 80% of trials fail to meet enrollment targets, with 19% terminated due to inadequate recruitment [10,11]. Ensuring a diverse, representative sample of participants is also a major priority. Coupling EHR data with portal-based recruitment has the potential for streamlining recruitment and engaging a large, representative pool of potential participants using a trusted and secure communication channel. Portal-based recruitment efforts have been described and widely implemented, but little is known about the patterns of responses among various patient populations and across different types of clinical research projects.

## Materials and methods

From June 2023 to July 2024, we utilized Epic MyChart for portal-based recruitment across 10 diverse research studies at Emory Healthcare. Emory adopted Epic as its EHR in October 2022, and most patients activated MyChart quickly due to billing system integration (61% of Emory patients had activated MyChart at the start of the pilot). This recruitment program was funded by the Georgia Clinical and Translational Science Alliance (CTSA) and approved by the Emory Institutional Review Board (IRB).

### Selection of research studies

The studies were carefully selected, each varying in inclusion/exclusion criteria, complexity, study type, and target sample size. The selection process was designed to ensure that the pilot program included a diverse spectrum of diseases, medical and procedural interventions, and demographic profiles (Supplementary Figure 1). Studies were also selected based on the compatibility of their entry criteria and design with the EHR format and technical capabilities, focusing on criteria available for constructing a computable patient phenotype. Each study obtained IRB approval before proceeding with the portal-based recruitment strategy.

### Recruitment process

Each study’s criteria were encoded into Structured Query Language using Epic Clarity, a data warehouse integrated into the healthcare organization’s EHR system. The query defines the source population as individuals who are at least 18 years old, alive, having an activated patient portal, active within the clinical setting, and having not opted out of research contact. Patients’ default contact preference within the system is “OK to Contact,” but patients may explicitly set their preference to “OK to Contact” or “Do Not Contact” at any time. The query is further tailored to each research study’s unique inclusion and exclusion criteria. Integrated into an EHR report, the query facilitates targeted outreach to eligible patients, who receive notifications and can express interest, decline, or not respond to the recruitment request. Query developers collaborate closely with the study team to ensure accurate representation of study criteria and to plan the number and frequency of invitations, based on recruitment needs and team capacity for follow-up. Recruitment processes are conducted within a secure, encrypted data environment, with access strictly limited to authorized personnel to protect patient data.

### Statistical analysis

Metrics on research invitations, responses, contact preferences, and timestamps are available in the data warehouse and are continuously monitored to ensure effective patient identification. Interval criteria refinement was occasionally required to improve specificity and outreach while balancing a need to preserve future engagement opportunities within the pool of patients who are “OK to Contact.” Analysis of participant responses – both broadly and in the context of each study type – and contact preferences over time was performed in Python and R. Simple descriptive statistics and univariable logistic regression were performed to explore differences in patient response rates and research participation interest among differing demographic groups.

## Results

### Overview of the source patient population in the healthcare system

Within the healthcare system, there are approximately 1.3 million active patients aged 18 and older; 23,700 were identified as potentially eligible for one of the 10 research studies and were invited to participate. Compared to the healthcare system source population, participants identified as potentially eligible for one of the research studies were more often female or black and had higher healthcare utilization, which was measured by the average number of encounters per year or number of active medications (Supplementary Figure 2). These differences are attributed to study-specific inclusion and exclusion criteria, which sometimes resulted in skewed demographics of potentially eligible participants. For instance, some studies were solely enrolling African Americans or females, thereby diverging from the overall population of the healthcare system.

### Response rates to research study invitations

Of the 23,700 patients who received a research study invitation, about 20% responded (either “Interested” or “Declined”), with 67% of respondents replying within 24 h. Patients of white race and individuals of non-Hispanic ethnicity were more likely to respond (non-Hispanic: OR = 1.9; white race: OR = 1.7; *p* < 0.0001 for both), and male patients were less likely to respond to recruitment invitations (OR = 0.86, *p* < .0001) (Table 1). Patients with higher healthcare utilization also had higher response rates. Those with at least five encounters per year were twice as likely to respond compared to those with 0–1 encounters, and individuals with more active medications were 60% more likely to respond (≥ 5 encounters per year: OR = 2.2; ≥ 5 medications: OR = 1.6; *p* < 0.0001 for both). Additionally, patients invited to interventional studies were 50% more likely to respond than those invited to observational studies (OR = 1.5, *p* < .0001).

**Table 1.** Characteristics of patients that received a study invitation

*n* (%)	Responded (*n*= 4,717, 20%)	No response (*n*= 18,975, 80%)	Odds ratio (95% CI)	*p*-value
Sex*				
Female	3,261 (21%)	12,487 (79%)	ref	
Male	1,455 (18%)	6,479 (82%)	0.86 (0.80, 0.92)	< 0.0001
Ethnicity*				
Hispanic	256 (13%)	1,768 (87%)	ref	
Non-Hispanic	4,075 (21%)	15,155 (79%)	1.9 (1.6, 2.1)	< 0.0001
Race*				
Black	2,234 (18%)	10,509 (83%)	ref	
White	2,016 (27%)	5,596 (74%)	1.7 (1.6, 1.8)	< 0.0001
Other	261 (14%)	1,644 (86%)	0.75 (0.65, 0.86)	< 0.0001
**Age**				
18–29	205 (17%)	973 (83%)	ref	
30–49	975 (24%)	2,139 (76%)	1.5 (1.2, 1.7)	< 0.0001
50–64	1,568 (17%)	7,452 (83%)	0.99 (0.85, 1.2)	0.99
65+	1,969 (21%)	7,411 (79%)	1.3 (1.1, 1.5)	0.044
**# Encounters/year**				
0–1	391 (12%)	2,785 (88%)	ref	
2–4	1,490 (17%)	7,158 (83%)	1.5 (1.3, 1.7)	< 0.0001
≥5	2,836 (24%)	9,032 (76%)	2.2 (2.0, 2.5)	< 0.0001
**# of Medications**				
0–1	766 (15%)	4,338 (85%)	ref	
2–4	959 (19%)	4,189 (81%)	1.3 (1.2, 1.4)	< 0.0001
≥5	2,992 (22%)	10,448 (78%)	1.6 (1.5, 1.8)	< 0.0001
Elixhauser comorbidity index – risk interpretation*				
Below average	511 (27%)	1,382 (73%)	ref	
Average/low	2,846 (20%)	11,244 (80%)	0.68 (0.61, 0.76)	< 0.0001
Moderate	769 (23%)	2,575 (77%)	0.81 (0.71, 0.92)	0.001
High	315 (24%)	987 (76%)	0.86 (0.73, 1.0)	0.076
**Study type**				
Observational	2,213 (17%)	10,886 (83%)	ref	
Interventional	2,504 (24%)	8,089 (76%)	1.5 (1.4, 1.6)	< 0.0001

*Note*: The odds ratio examines the odds of responding to the study invitation depending on the demographic, with the reference group (ref) serving as the baseline for comparison. Percentages are based on row totals.

*Missing data are due to individuals with unknown, other, or missing values.

### Interest rates among respondents

Among the 20% of patients who responded to their research invitation, 56% expressed interest in joining the study (Table 2). Males showed greater interest, and white individuals showed lower interest compared to their gender/racial counterparts (male: OR = 1.2, *p* = 0.019; white race: OR = 0.85, *p* = 0.008). High healthcare utilization and medication (≥ 5 encounters per year or medications) were also associated with increases in interest, compared to those with fewer encounters or medications (≥ 5 encounters per year: OR = 1.3, *p* = 0.024; ≥ 5 medications: OR = 1.4, *p* < .0001). Patients invited to interventional studies were more likely to express interest in joining the research (OR = 1.4, *p* < .0001).

**Table 2.** Characteristics of patients that responded to their study invitation

*n* (%)	All (*n*= 4,717)	Interested (*n*= 2,636, 56%)	Declined (*n*= 2,081, 44%)	Odds ratio (95% CI)	*p*-value
Sex*					
Female	3,261	1,785 (55%)	1,476 (45%)	ref	
Male	1,455	850 (58%)	605 (42%)	1.2 (1.0, 1.3)	0.019
Ethnicity*					
Hispanic	256	149 (58%)	107 (42%)	ref	
Non-Hispanic	4,075	2,290 (56%)	1,785 (44%)	0.92 (0.71, 1.2)	0.53
Race*					
Black	2,234	1,312 (58%)	922 (41%)	ref	
White	2,016	1,103 (55%)	913 (45%)	0.85 (0.75, 0.96)	0.008
Other	261	115 (44%)	146 (56%)	0.55 (0.43, 0.72)	< 0.0001
**Age**					
18–29	205	139 (68%)	66 (32%)	ref	
30–49	975	648 (67%)	327 (34%)	0.94 (0.68, 1.3)	0.71
50–64	1,568	914 (58%)	654 (42%)	0.66 (0.48, 0.90)	0.010
65+	1,969	935 (48%)	1,034 (53%)	0.43 (0.31, 0.58)	< 0.0001
**# Encounters/year**					
0–1	391	203 (52%)	188 (48%)	ref	
2–4	1,490	789 (53%)	701 (47%)	1.0 (0.83, 1.3)	0.72
≥5	2,836	1,644 (58%)	1,192 (42%)	1.3 (1.0, 1.6)	0.024
**# of Medications**					
0–1	766	386 (50%)	380 (50%)	ref	
2–4	959	489 (51%)	470 (49%)	1.0 (0.8, 1.2)	0.80
≥5	2,992	1,761 (59%)	1,231 (41%)	1.4 (1.2, 1.7)	< 0.0001
Elixhauser comorbidity index – risk interpretation*					
Below average	511	318 (62%)	193 (38%)	ref	
Average/low	2,846	1,540 (54%)	1,306 (46%)	0.72 (0.59, 0.87)	0.0007
Moderate	769	435 (57%)	334 (43%)	0.79 (0.63, 0.99)	0.044
High	315	190 (60%)	125 (40%)	0.92 (0.69, 1.2)	0.58
**Study type**					
Observational	2,213	1,139 (52%)	1,074 (49%)	ref	
Interventional	2,504	1,497 (60%)	1,007 (40%)	1.4 (1.2, 1.6)	< 0.0001

*Note*: The odds ratio examines the odds of interest in the study depending on the demographic, with the reference group (ref) serving as the baseline for comparison. Percentages are based on row totals.

*Missing data are due to individuals with unknown, other, or missing values.

### Impact of research study invitations on patient contact preferences

**Figure 1.**
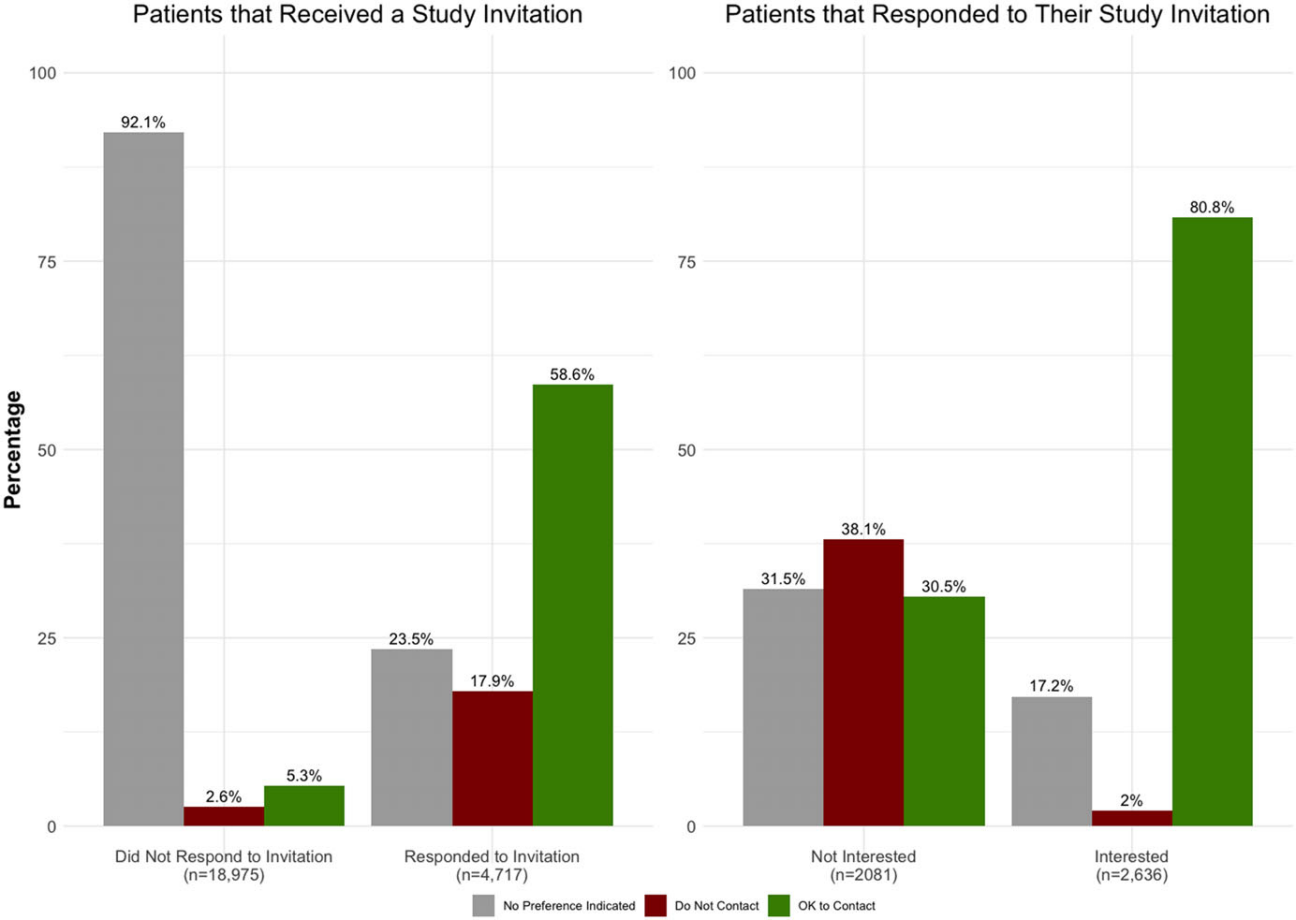
Patient contact preference after receiving a research invitation by patient response and interest to the research study. *Note*: Percentage of contact preference is per response/interest group. Contact preference is the most recent preference individuals made after receiving a research invitation. Only individuals with a contact preference of “OK to Contact” or “No Preference Indicated” were sent an invitation to begin with.

Receiving a research invitation prompted some patients to update contact preferences. Among those who responded to an invitation, 59% chose to explicitly set their status to “OK to Contact” for future research contact (Figure 1). In contrast, 92% of patients who did not respond left their contact preference as the default “No Preference Indicated.” Notably, 81% of respondents who expressed interest in the study subsequently changed their preference to “OK to Contact.” Among the roughly 2,000 individuals who declined the study invitation, 38% changed their contact preference from the default to “Do Not Contact.”

## Discussion

### Key findings and implications for recruitment practice

This study offers valuable insights into the effectiveness of EHR systems and patient portals as tools for recruiting participants in clinical research. By engaging with patients directly through a trusted digital platform, our recruitment strategy efficiently reached a broad and diverse patient population. An overall response rate of 20%, with the majority of responses occurring within the first 24 h, underscores the accessibility of patients via portals and their likelihood of responding promptly. Furthermore, the substantial interest among respondents (56%) highlights the potential effectiveness of this approach.

Several factors influenced patient responses and their interest in participating in research. While there was interest across demographic groups, males and Black individuals had lower overall response rates. This aligns with findings from other studies, which also show that Black individuals and males are less likely to respond to invitations or engage with patient portals [12,13]. Interestingly, when these underrepresented groups did respond, they demonstrated higher odds of being interested in research through patient portal recruitment, suggesting a shift from traditional recruitment methods [12,14]. Importantly, while there are differences in overall response and study interest rates, these differences are not of a magnitude that diminishes the value of portal-based recruitment as an effective tool or raises significant concern about skewing populations. Such differences may vary depending on specific study features and eligibility criteria. Nonetheless, it is crucial to continue examining variations in response patterns across different groups to identify the most effective use of this strategy, to address potential disparities or gaps in response, and to maximize representativeness in enrollment. Additionally, these findings highlight the need for studies that explore how different types of messaging might influence response patterns.

Higher healthcare utilization – evidenced by frequent encounters and medication use – emerged as a significant predictor of both response and interest in research participation. Patients who interact more regularly with the healthcare system may feel more comfortable and trusting of the institution; they also may be more invested and interested in contributing to research that has the potential to enhance their care. Interestingly, the number of patient comorbidities did not show a strong correlation with overall response or study interest rates, suggesting that engagement may be driven more by familiarity with the healthcare system than by health status alone. This finding is not altogether surprising, but it underscores the importance of familiarity and comfort with a healthcare system for recruitment efforts. Exploration of strategies to help increase familiarity with or awareness of research among those who “touch” the system less is an important priority in engaging broader populations.

When using the portal for research recruitment, these data highlight several key considerations. First, not all inclusion and exclusion criteria can be captured directly from the Epic database. Additional steps, such as administering questionnaires or conducting detailed chart reviews, will still be necessary to confirm patient eligibility in many cases, and the role of EHR-based tools to facilitate that process warrants further study. Second, recruitment messages should be carefully tailored to each study, using clear and appropriate language that aligns with the study’s goals and resonates with the target audience. Continued exploration and assessment of the impact of various message types will be important. Finally, as most participants express interest within a day of receiving an invitation, timely follow-up is crucial. Delayed responses risk losing interest, making prompt engagement essential when planning message frequency and volume.

### Strengths and limitations

This patient portal recruitment program demonstrated notable strengths. It benefited from a large sample size within the hospital system, enabling robust data collection and analysis. Additionally, the program sourced patients from multiple hospital sites, enhancing geographical and demographic diversity. The range of diverse research studies, each with differing study designs and criteria, further enriched the program’s scope and applicability. Furthermore, the nature of the recruitment method is efficient and convenient, enables timely communication, and enhances EHR data integration.

There are several limitations to consider. Portal-based recruitment has inherent challenges, including the fact that not all patients use the portal regularly, which could introduce demographic bias related to socioeconomic factors or digital literacy [2]. Additionally, variations in study-specific criteria – such as medication, diagnoses, race, and age – may lead to over- or under-representation of certain demographic groups in analysis [15]. This recruitment program also had a limited number of studies, and it was the first time patients in this healthcare system were exposed to portal-based invitations; it is unclear whether response rates will change with repeated exposure. Lastly, we were unable to track how many of these responses ultimately resulted in enrollment.

## Conclusion

Integrating EHR and portal-based recruitment methods holds significant promise for enhancing the efficiency and reach of clinical research recruitment. By leveraging these digital platforms, researchers can streamline recruitment processes, promote participant engagement, and cultivate a more representative and diverse study population. Optimizing the use of these technologies will be vital in overcoming existing recruitment barriers and advancing clinical research outcomes within the healthcare system.

## Supporting information

Schwinne et al. supplementary material Schwinne et al. supplementary material

## Supplementary material

The supplementary material for this article can be found at https://doi.org/10.1017/cts.2024.692.
