# Supplementary material for: Transforming research recruitment: Leveraging EHR systems and patient portals
Source: J Clin Transl Sci. 2024 Dec 26;9(1):e10. doi: 10.1017/cts.2024.692 (PMC11795853; doi:10.1017/cts.2024.692)
Supplement: Schwinne et al. supplementary material [file S2059866124006927sup001.docx]

**SUPPLEMENTARY MATERIALS**

*Supplementary Figure 1. Brief Description of the 10 Research Studies Utilizing MyChart Recruitment*

| **Research Study** | **Study Type** | **Main Criteria** | **Criteria Complexity** | **~# of Invites Sent** |
| --- | --- | --- | --- | --- |
| 1 | Observational | HI 50-75 y/o | Simple | 5600 |
| 2 |  | NMNC |  | 2700 |
| 3 |  | Females ≥ 45 y/o with coronary artery disease | Moderate | 1700 |
| 4 |  | Postpartum and RSV vaccinated |  | 1700 |
| 5 |  | NMNC |  | 700 |
| 6 | Interventional | Peripheral Artery Disease and on statins | Complex | 2000 |
| 7 |  | Coronary Artery Disease with a bypass procedure, and see explicit providers |  | 2600 |
| 8 |  | Long Covid Diagnosis | Moderate | 750 |
| 9 |  | Black individuals ≤ 65 y/o with PTSD |  | 2600 |
| 10 |  | HI not on statins |  | 1000 |

Note: HI = Healthy Individuals with no major conditions; NMNC = no major neurological conditions

*Supplementary Figure 2. Demographics of Individuals in Healthcare System and Those Further Identified for Research*


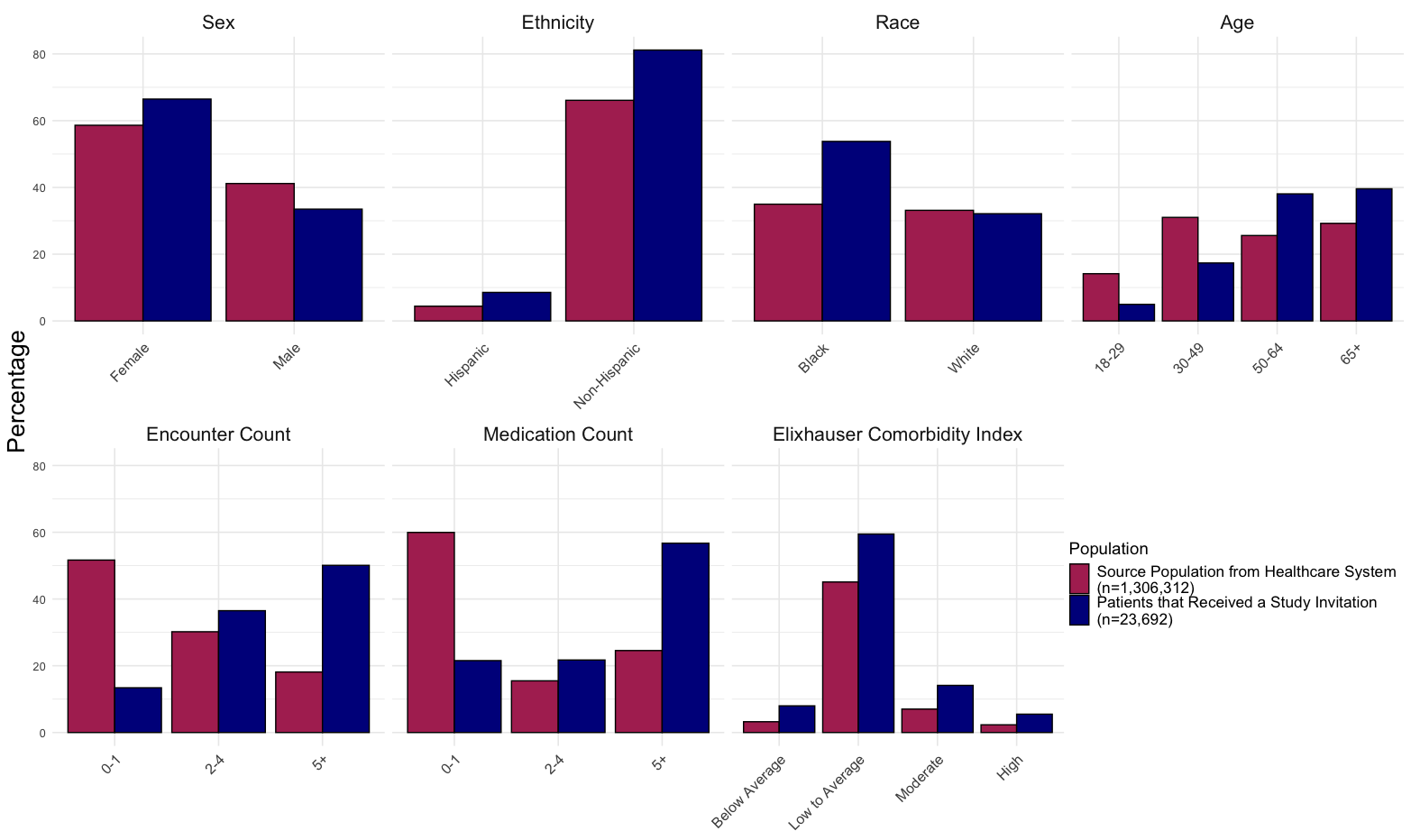
Note: All individuals identified for research come from the healthcare system. Sex, ethnicity, and race demographics have missing data, especially in the healthcare system population. Percentages represent the proportion of individuals in the demographic group out of the total number of individuals in the population (healthcare system or those who were identified to be potentially eligible for a research study and were then sent a research invitation
